# Gut Microbial Influences on the Mammalian Intestinal Stem Cell Niche

**DOI:** 10.1155/2017/5604727

**Published:** 2017-08-22

**Authors:** Bailey C. E. Peck, Michael T. Shanahan, Ajeet P. Singh, Praveen Sethupathy

**Affiliations:** ^1^Department of Surgery, School of Medicine, University of Michigan, Ann Arbor, MI 48105, USA; ^2^Department of Biomedical Sciences, College of Veterinary Medicine, Cornell University, Ithaca, NY 14853, USA

## Abstract

The mammalian intestinal epithelial stem cell (IESC) niche is comprised of diverse epithelial, immune, and stromal cells, which together respond to environmental changes within the lumen and exert coordinated regulation of IESC behavior. There is growing appreciation for the role of the gut microbiota in modulating intestinal proliferation and differentiation, as well as other aspects of intestinal physiology. In this review, we evaluate the diverse roles of known niche cells in responding to gut microbiota and supporting IESCs. Furthermore, we discuss the potential mechanisms by which microbiota may exert their influence on niche cells and possibly on IESCs directly. Finally, we present an overview of the benefits and limitations of available tools to study niche-microbe interactions and provide our recommendations regarding their use and standardization. The study of host-microbe interactions in the gut is a rapidly growing field, and the IESC niche is at the forefront of host-microbe activity to control nutrient absorption, endocrine signaling, energy homeostasis, immune response, and systemic health.

## 1. Introduction

**Figure 1:**
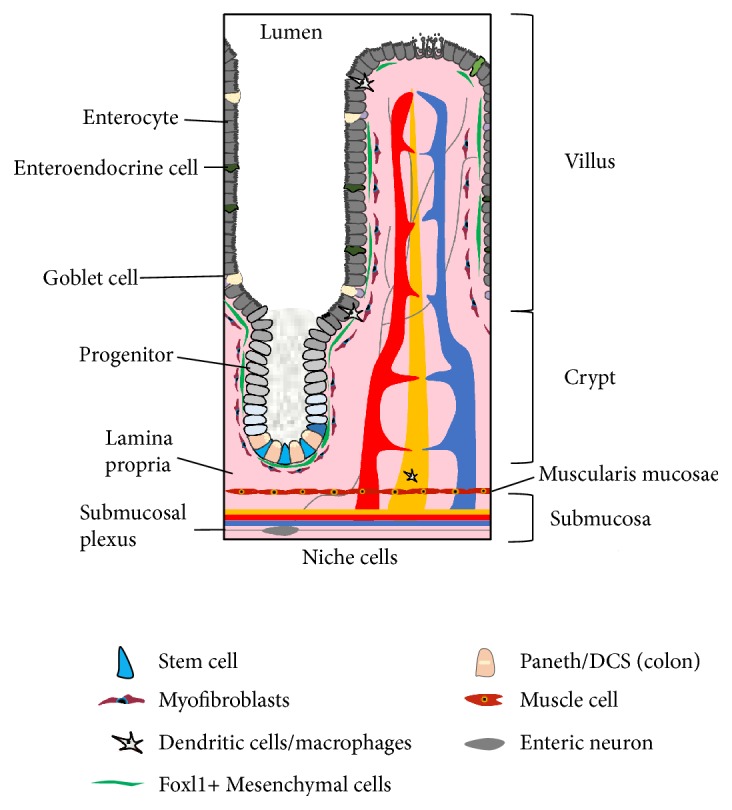
The intestinal stem cell niche. Intestinal stem cells have the capacity to generate, via a population of progenitor cells, all differentiated cell types of the intestinal epithelium including enterocytes, goblet cells, Paneth cells, and enteroendocrine cells. Those cell types that are known or suspected to comprise the intestinal stem cell niche include the adjoining Paneth cells of the small bowel, or the deep crypt secretory cells of the colon, as well as myofibroblasts, dendritic cells, macrophages, muscle cells, and enteric glia and neurons found in the subepithelial lamina propria and submucosal compartments of both small and large intestine.

The gastrointestinal (GI) tract is the primary site of nutrient absorption and digestion, a barrier to harmful toxins and pathogens, and the largest endocrine organ of the body involved in the maintenance of metabolic homeostasis. The intestinal epithelium comprises the innermost monolayer of cells in the GI tract that directly interfaces with the gut lumen and is replaced every 2-3 days in mice and 3–5 days in humans [1–3]. The monolayer is organized by units of villi (projections into the lumen) and crypts (invaginations into the lamina propria—connective tissue and immune cells that reside beneath the epithelial layer; see Figure 1). The villi contain specialized, differentiated cell types including cells of the absorptive lineage (e.g., enterocytes) and of the secretory lineage (e.g., enteroendocrine cells and goblet cells) [4]. The rapid renewal of these cells is driven by actively proliferating intestinal epithelial stem cells (IESCs) that reside at the base of the crypt in a functionally defined niche that includes epithelial Paneth cells as well as nearby nonepithelial cell types including immune cells of the lamina propria and stromal cells. The delicate balance in IESCs between self-renewal and differentiation controls intestinal epithelial homeostasis and regeneration, particularly in response to injury, inflammation, or altered microenvironment. The niche in which IESCs are embedded helps maintain this balance. In addition to the cell types mentioned above, microbiota residing in the intestinal lumen are key members of the IESC niche.

The intestine is a suitable environment for the habitation of a high density of microbes (>100 trillion bacteria, viruses, fungi, archaea, and protists) [5–9]. These resident microbes take part in a complex triangular ecological niche involving nutrients and host cells [5–7]. It is important to note, however, that the niche, much like the overall cellular composition, is nonuniform across different anatomical and functionally-distinct regions of the intestine, including the duodenum, jejunum, ileum, caecum, and colon. These different intestinal segments exhibit varying microbial density and composition and are subject to different nutritional and environmental exposures [8, 9]. Together with neighboring host cells, the microbiota influence niche functions, and thereby modulate IESC behavior differently across the length of the intestine [10]. As such, it is important to consider regional differences in microbial composition that may contribute to different functions when studying the IESC niche. In what follows, we will provide an overview of the major cell types in the IESC niche and then a more detailed description of the known contributions of resident microbiota.

## 2. The Cell Types of the Intestinal Epithelial Stem Cell Niche

### 2.1. Intestinal Epithelial Stem Cells

The intestinal crypt in which IESCs reside harbors some IESCs-derived cell populations, including transit-amplifying progenitor cells, enteroendocrine cells (EECs), and Paneth cells [3, 11]. Under normal conditions, IESCs predominantly divide symmetrically [12, 13]. Certain stress contexts can trigger asymmetric division in order to prevent the hyperabundance of IESCs [14]. IESCs produce transit-amplifying progenitor cells that divide very rapidly (approximately every 12 hours) and comprise two-thirds of the base of the crypt. They progressively differentiate into various specialized intestinal epithelial cells (e.g., enterocytes) that generally migrate up the crypt-villus axis [12]. Once these differentiated cells reach the apex of the villus, they undergo anoikis (a form of programmed cell death, where cells detach from the extracellular matrix) and are released into the lumen of the intestine [15, 16]. Paneth cells and a subset of EECs represent exceptions to this pattern, as these cells can migrate downward toward the base of the crypt where IESCs reside, forming a part of the IESC niche. Paneth cells also have an increased lifespan relative to other differentiated cell lineages, estimated to be greater than 3-4 weeks before undergoing anoikis [17, 18]. And, while there are conflicting reports, some types of enteroendocrine cells may also survive longer than absorptive enterocytes [19, 20].

**Figure 2:**
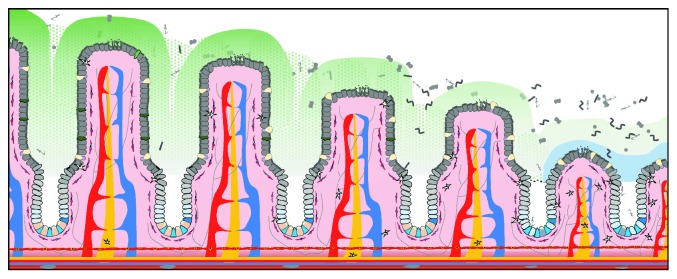
Regional differences along the small intestinal tract. The proximal-distal axis of the small intestine displays a gradient of various properties. Not only are mirobial loads progressively loads increased toward the ileal end of the intestine, but villus length gradually decreases in this same direction as well. The mechanisms by which luminal microbes could affect such changes in intestinal architecture may involve TLR activation, extracellular vesicles (EVs), metabolic byproducts, and/or other heretofore unspecified direct and indirect on intestinal epithelial stem cells.

Crypt size, proliferative index, and the distribution of proliferative cells within the crypt are variable across the intestinal tract (see Figure 2, [8]). This type of regional variability is not uncommon in other organ systems with adult multipotent stem cells [21–23]. The actively cycling IESCs of the small intestine are located in the crypt base and are marked by high expression of several genes including *Lgr5*, *Olfm4*, and *Ascl2*, as well as by low expression of *Sox9* [24]. Slower cycling or reserve IESCs are marked by high expression of *Bmi1*, *Tert*, *Hopx*, *Lrig1*, and *Sox9*. However, these markers are not specific, as several of them are also found in actively cycling IESCs (e.g., *Lrig1*) or EECs (e.g., *Sox9*, [25]). Particularly fascinating is the observation that some secretory and absorptive progenitors exhibit plasticity; that is, the potential to revert back to IESCs in response to injury [26–29], suggesting that the reserve stem cell population is broader and less defined than certain differentiated IEC populations (also see reviews [30, 31]). Recent single-cell transcriptomic work has shown that there is heterogeneity even among Lgr5+ actively cycling IESCs [32]. This molecular heterogeneity was also seen in earlier studies comparing populations of CD24^lo^ and side-population IESCs [33], as well as in a very recent RNA-seq-based comparison of IESC populations isolated by diverse methods [34].

The chromatin state, and many transcription factors and signaling cascades, regulate IESC stemness. The position of the IESC within the crypt is a major determining factor of its self-renewal capacity, driven in part by Wnt and Delta-Notch signaling [12, 35]. Transcription factors such as Klf5, Gata4, Gata6, Ascl2, and Yy1 have been shown to control intestinal stem cell fate, and their deficiency causes disruption of intestinal architecture [14, 36]. More recently, microRNAs too have emerged as key regulators of the niche and responders to environmental stimuli in IESCs [37, 38]. For example, miR-375 in murine IESCs is highly sensitive to the presence of microbes, and loss-of-function studies in ex vivo mouse enteroid cultures suggest that it may be a prominent regulator of intestinal epithelial proliferation [38]. For further detailed review of IESCs, we refer the reader to recent review article [39].

### 2.2. Paneth Cells

Paneth cells are epithelial cells of the small intestine that are located between and around IESCs and take part in shaping the crypt microenvironment and regulating microbial interactions within the crypt by secreting antimicrobial peptides [40]. They are present throughout the entire small intestinal tract, and they increase in number along the proximal-distal axis. Unlike villus epithelial cells that get replaced every 3–5 days, the life span of Paneth cells in the crypt is about 30 days [40]. As part of their role in the niche, they also release growth factors that directly influence the neighboring IESCs [40], cementing their role in the niche. Under environmental stress, Paneth cells act to protect and stimulate IESCs. For example, under conditions of caloric restriction, luminal cyclic adenosine diphosphate (cADP) derived from Paneth cells induces IESC. Interestingly, however, ablation of Paneth cells *in vivo* does not appear to impact IESC proliferation and differentiation [41] or the distribution of microbes within the gut [17], possibly due to compensatory responses by other niche cells [41]. Loss of Paneth cells has been shown to compromise the barrier integrity of the intestinal epithelium [42]. Recent work has suggested that the large intestine may also harbor Paneth cell-like deep crypt secretory (DCS) cells [43]. More work is needed however to evaluate these cells further and determine the extent to which they contribute to colon IESC niche functions [43]. For further detailed descriptions of Paneth cells, we refer the reader to the following reviews [44, 45].

### 2.3. Enteroendocrine Cells

Enteroendocrine cells (EECs) are occasionally located within the crypt and play a vital role in gut physiology and may contribute to the IESC niche microenvironment [46, 47]. Though EECs make up less than 1% of all intestinal epithelial cells, they have an important function in sensing the luminal environment (nutrients, bile acids, microbes, etc.) and secreting hormones, including Glp-1, Cck, Pyy, Gip, ghrelin, and neurotensin, in order to coordinate systemic energy regulation [48, 49]. There are many different subtypes of EECs based on the hormones that they most readily express and secrete. For example, both I cells and K cells are EEC subtypes that reside predominantly in the proximal small intestine, but secrete the hormones CCK and GIP, respectively, which have different endocrine effects [50, 51]. The abundance and types of EECs vary throughout the gastrointestinal tract; some EEC subtypes are found throughout the small and large intestine (e.g., N cells: neurotensin-secreting EECs), whereas others are found primarily in the small intestine (e.g., K cells, I cells, and S cells: gastric inhibitory peptide-, cholecystokinin-, and serotonin-secreting EECs, resp.) [50]. EECs are also abundant in the rectum, where they are found at the highest frequency in the GI tract other than the proximal small bowel [52–54]. It has been suggested that crypt EECs, or possibly secretory progenitor cells in general, may comprise a reserve pool of IESCs that actively proliferate in response to intestinal injury [25, 49, 55, 56]. Their contributions to the maintenance of IESC function remain poorly characterized; however, it is known that certain EEC-secreted peptides, such as Glp-2, can serve as paracrine signaling molecules to promote intestinal epithelial proliferation [57]. EECs have also been shown to respond to microbe-derived peptides and therefore may act as a conduit signaling mechanism for the IESC niche [58]. For example, recently it was shown that colonic exposure to proteins from *Escherichia coli* stimulate Pyy and Glp-1 release from EECs in rats [59]. Much more work remains to be done in order to define more rigorously the functional importance of crypt EECs to the IESC niche.

### 2.4. Stromal Cells

In the adult intestine, the epithelium is surrounded by stromal cells of the mesenchymal lineage. These cells facilitate intercellular crosstalk through several factors that regulate IESC proliferation and differentiation and therefore are considered an integral aspect of the IESC niche. Subepithelial mesenchymal stromal cells produce bone morphogenetic proteins (BMPs), which are members of the TGF-*β* superfamily that antagonize Wnt signaling along the crypt-villus axis, thereby inhibiting IESC expansion and promoting epithelial cell differentiation [60]. Other mesenchymal cells including myofibroblasts secrete BMP inhibitors that promote Wnt-mediated IESC self-renewal [61]. Recently, a seminal study by Aoki et al. and Kaestner and colleagues described a small population of elongated *Foxl1*-expressing mesenchymal cells that envelop both the crypts and villi of the intestinal epithelium and produce a number of growth factors including those of the Wnt and Bmp family to support IESCs [62]. Ablation of these cells, but not other niche cells like Paneth cells, results in severely compromised crypt proliferation. These data suggest that the *Foxl1*+ mesenchymal cell population constitutes an essential component of the IESC niche [35]. In sum, the entire collection of subepithelial stromal cells mediates an intricate signaling network that maintains balance between IESC self-renewal and differentiation along the crypt-villus axis. Comprehensive characterization of the functional diversity of mesenchymal cells and their roles in the niche remains an active and important area of research.

Macrophages are crucial sentinels in the healthy intestinal lamina propria that are required for maintenance of intestinal homeostasis in the face of microbiota and food antigens [63]. Epithelial tuft cells and goblet cells mediate immune response to microbes and microbial-derived peptides by secreting chemokines to which these intestinal macrophages readily respond [64–68]. In both rodents and humans, intestinal macrophages are more numerous in the small intestine than in the large bowel. It is increasingly being recognized that macrophages, in addition to serving an innate immune function, can regulate intestinal stem cell function. Recently, Saha et al. found that radiation-induced intestinal injury is ameliorated by enhanced stem-cell proliferative function stimulated in part by macrophage-secreted Wnt factors [69].

### 2.5. Enteric Nervous System

The enteric nervous system (ENS) plays a vital role in many aspects of GI tract function, including orchestrating peristalsis and fluid secretion required for food digestion and nutrient absorption and sustaining a healthy luminal microbiome. Also, it has been found that the ENS can influence IESC function. For example, Lundgren et al. have shown that modification of mucosal afferent nerve function modulate IESC proliferation [70]. Given that enteric nerve cells act synchronously with clonally related neurons, the effect may be broadly translated across multiple crypts [71]. Moreover, in addition to their neural support roles, glial cells of the ENS also contribute to intestinal epithelial proliferation and repair after injury through the secretion of proepidermal growth factor (pro-EGF) [72, 73].

For detailed reviews of the diverse cells types within the IESC niche, see [11, 74, 75].

## 3. Role of Gut Microbiota in the Stem Cell Niche

To maintain gut homeostasis and proper function, IESCs must respond either directly or indirectly to apical luminal and basolateral abluminal factors, most notably gut microbiota and dietary components. Cells of the IESC niche have evolved a number of mechanisms to manage a constantly changing luminal microenvironment. Constituents of the intestinal microbiota and their products are potentially highly potent regulators of IESC activity due to their proximity to the intestinal epithelia, as well as their profound effects on host nutrition, metabolism, and mucosal barrier integrity.

### 3.1. Region-Specific Roles for Gut Microbiota in the Control of Intestinal Epithelial Renewal

Decades of research on murine models has revealed that luminal bacteria can shape a variety of morphological and functional features of different intestinal regions and cellular subpopulations. One of the oldest observations was made in the 1960s through studies of germ-free (GF) and antibiotic-treated mice and rats. It was noted that these rodents exhibited decreased villus height and crypt depth in the jejunum and ileum, increased villus height and decreased crypt depth in the duodenum, reduced mucosal surface area, lowered mitotic indices, reduced lamina propria volume, and slower transepithelial migration rates compared to conventionally raised (CR) animals [76–84]. These findings were suggestive of one or more of several possibilities. For example, shorter crypts could be indicative of decreased proliferation, and/or increased/premature differentiation, and/or progenitor apoptosis. Subsequent follow-up studies have evaluated these possibilities and are shedding light on the context-specific effects of colonization on intestinal physiology. Current state-of-the-art follow-up studies include whole transcriptome profiling (both aggregate and single-cell) and fluorescent immunohistochemistry for markers of active proliferation and apoptosis. For example, we recently demonstrated that genes in pathways associated with mitotic cell cycle are transcriptionally upregulated in jejunal cell populations enriched for stem cells of conventionalized animals relative to GF animals [38]. Yu et al. also demonstrated increased ileal crypt proliferation in ex-germfree mice in response to colonization with microbiota from healthy infants relative to colonization with microbiota from infants with low weight gain [85]. In studies in which GF rodents were exposed to commensal microbes, increased colonic epithelial proliferation and deepened large bowel crypts were observed [86, 87]. Although this effect was reported in other small intestinal regions as well, it was evident that the magnitude of the effect of microbes on epithelial morphology is region-specific [88, 89]. Duodenal and jejunal intestinal epithelia from CR mice display slightly increased proliferation relative to ileum, despite the fact that duodenal and jejunal luminal bacterial loads are substantially less than what is found in the ileal lumen [90]. The potential primacy of microbial composition over total bacterial number on the control of intestinal epithelial proliferation was demonstrated by the observation that exposure to specific bacterial species such as the breast milk-derived probiotic strain *Lactobacillus reuteri* DSM 17938 induces intestinal epithelial proliferation while other strains like *L. reuteri* PTA 6475 do not [91]. Viruses may also contribute to overall intestinal epithelial morphology and physiology. For example, certain strains of murine norovirus can modulate innate immunity and mediate some negative effects on the intestinal epithelium of dextran sodium sulfate and certain antibiotic treatments [92].

### 3.2. Mechanisms of Microbial Influence on IESCs

Although it is clear that the presence of luminal microbes is correlated with structural and functional changes in IECs, it is often difficult to determine whether microbes or the experimental treatments that induce microbial changes are responsible for these effects. Modifications of diet and antibiotic treatments have been employed historically to alter the intestinal microbiota in order to study host effects. However, identifying the precise, and likely multiple, mechanisms by which microbiota influence the IESCs has proven challenging especially given the regional specificity and diversity of microbes and their derived metabolites. Regulation of IESCs by microbiota may occur either through direct and or indirect means, and understanding mechanisms of niche-microbe interactions has therapeutic relevance. Secreted factors that stimulate the Wnt/*β*-catenin signaling pathway are the primary means by which the niche offers support for IESCs. For example, following injury from radiation, mesenchymal stem cells activate the Wnt/*β*-catenin signaling pathway and support Lgr5^+^ stem cell growth to promote regeneration [93]. Similarly, as mentioned above, Saha et al. demonstrated that macrophages secrete Wnt factors in exosomes to support the intestinal stem cell niche during regeneration and protect it from radiation-induced injury [69]. Yet, the extent to which these and other niche cells act in response to changes in the gut microbiota during homeostasis or following injury has not been fully elucidated.

#### 3.2.1. Potential Mechanisms of Direct Influence

The intestinal stem cell niche has been described as being maintained under completely sterile conditions in the absence of injury [94–96]. However, microbes residing within intestinal mucosa, and indeed within healthy intestinal crypts, are well documented, which raises the possibility of direct regulation of intestinal stem cell physiology by gut microbiota. The earliest visualization of microbiota in direct contact with the intestinal epithelium was in the 1970s using scanning electron microscopy on mouse intestine. These studies showed microbes attached to the openings of the crypts of Lieberkühn via long webbing filaments [97–100], and not fully separated from the epithelium by the mucus layer. However, it was not until recently that microbes were visualized deep within crypts [101, 102]. One main challenge in identifying these crypt-based microbes stems from the use of common washing and fixation methods that dissolve or disturb microbial biofilms and host-mucins [98, 103]. Using a fixation method that preserves the biofilms, such as anhydrous Carnoy's fixative, together with extremely cautious sectioning techniques, has further improved visualization of microbes within intestinal crypts [5, 95, 99, 101, 102]. Current research suggests that crypt-based microbes are found primarily in the colon and caecum, which is consistent with the overall microbial density gradient within the gut [5, 101, 102]. Bacterial species found within the crypt, as identified by 16S sequencing, and fluorescent in situ hybridization (FISH) of murine colonic crypts, indicate the predominance of bacteria capable of aerobic metabolism, including species of *Acinetobacter* and *Proteobacteria* [5]. This finding is interesting given that the flora of the small intestine is also enriched for aerobes [104, 105]. Following GI infection, certain pathogenic microbes have been found to more frequently occupy the crypt niche, even in the upper GI tract, and it has been suggested that colonization of the crypts might promote pathogenic longevity leading to chronic infections, as is seen with *Helicobacter pylori* in the stomach [106]. On the other hand, the presence of residing *H. pylori* in gastric crypts also prevents secondary infections, a form of “colonization resistance,” which may be beneficial to the host's health.

Less well studied is the possibility that microbiota may stimulate IESCs directly through the release of outer membrane vesicles (OMVs). Given that IESCs take up macrophage-derived exosomes [69], much like what has been observed in enterocytes, it is possible that IESCs also take up outer membrane vesicles (OMVs) produced by gram-negative bacteria localizing at the base of villi or within the crypts [107–109]. OMVs are similar in size to exosomes and are taken up via similar pathways, such as through caveolin or lipid raft-mediated endocytosis [110]. OMVs may carry bacterially derived and molecularly active peptides, virulence factors, small RNAs, and DNA, all of which could act to modify IESC gene expression patterns. Uptake of OMVs by IESCs has not been formally evaluated, though uptake of OMVs by other intestinal epithelial cells has been demonstrated [111]. This may suggest multiple possibilities by which microbes directly regulate IESC gene expression and cellular behavior.

#### 3.2.2. Potential Mechanisms of Indirect Influence

Resident microbiota, as part of the symbiotic relationship with humans, metabolize and ferment foods in the intestinal lumen. Byproducts and metabolites from these processes can be absorbed or act as receptor ligands by both the host as well as by other microbes within the gut. Some of the most widely studied microbial metabolites include short-chain fatty acids (SCFAs), which are produced primarily in the colon through the fermentation of dietary fibers. Kaiko et al. found that SCFA butyrate suppressed colonic stem cell proliferation [112], perhaps through receptors encoded by *Ffar3*, *Ffar2*, and *Niacr1* [113]. *Ffar2* is robustly expressed in mouse jejunal IESCs and is downregulated upon conventionalization [38]. Importantly, enterocyte metabolism of butyrate at the entrance of the colonic crypt was an important modulator of the SCFA dosage received by IESCs, suggesting that certain enterocytes may support the niche [114]. However, given the trace amounts of SCFAs in the small intestine, this may not be a prominent pathway regulating the small bowel IESC niche. In this same screen by Kaiko et al., nicotinic acid (or niacin) was found to have pro-proliferative effects on colonic stem cells [112]. Niacin is ingested or biosynthesized by the gut microbiota [115] and may therefore be a strong candidate for regulation of small intestine IESCs. Further research into small intestinal metabolites that regulate IESCs is warranted.

Microbial stimulation of non-IESC niche cells may result in the secretion of signaling peptides that in turn influence IESC physiology. For example, Paneth cells of the small intestine (and possibly DCS cells of the colon) form a major component of the IESC niche. They secrete a number of antimicrobial peptides and growth factors including lysozyme, *α*-defensins, WNT, EGF, and Notch to their neighboring stem cells, and when dysregulated leave the host more susceptible to infection and other physiological abnormalities (see reviews [40, 116–118]). TLR activation in Paneth cells is associated with the degranulation and secretion of defensins into the crypt [119–122], which would modulate the niche microenvironment. However, it is not yet clear what the precise effect of Paneth cell degranulation is on IESC physiology.

Other niche cells may provide more insight, though in some cases their actions on IESCs may be interdependent. Niche cells respond to various microbial signals (e.g., via TLR receptors) and metabolites (e.g., SCFA), resulting in a number of downstream stimuli that could alter IESC physiology. Some EECs, such as L-cells located along the crypt-villus axis, release Pyy and Glp-1 in response to microbial stimuli [119]. Pyy in turn stimulates intestinal epithelial proliferation and differentiation both *in vivo* and *in vitro* [123, 124]. EECs that reside outside the niche may also contribute toward the control of IESC behavior by serving as intermediates in multicellular signaling pathways initiated by resident microbes. Tuft cells have recently been shown to respond in part to parasites and helminthes by secreting IL-25 [125]. IL-25 induces innate lymphoid cells to secrete the IESC stimulating factor IL-13 [67], resulting in increased goblet and tuft cell differentiation [65]. IL-33 expression in intestinal stromal cells provides another possible mechanism by which microbes may regulate IESCs, as some microbes, including helminths and other parasites, induce IL-33 release from lymphocytes [126]. For example, it was recently shown that TNF-*α*- and IL-1*β*-stimulated IL-33 release from pericryptal fibroblasts in response to *Salmonella typhimurium* infection promotes secretory cell differentiation of IESCs [127]. Sources of IL-33 are not limited to these fibroblasts; epithelial cells may also express IL-33 thereby further regulating IESC differentiation.

Finally, microbiota-derived neurostimulatory peptides, including glutamate, serotonin, and GABA, as well as macronutrients like glucose and fatty acids, can act as neurotransmitters to stimulate the enteric nervous system, which in turn can regulate IESC function (see reviews Mazzoli and Pessione [58] and Neunlist and Schemann [128]).

## 4. Tools to Study Microbiota Interactions in the IESC Niche

A number of questions remain with regard to how the microbiota may influence the IESC niche. Over the past several decades, experimental models have been developed, which span *in vitro*, ex vivo, and *in vivo* methodologies (Table 1). Here, we touch on the most recently developed as well as the most widely used tools for studying IESC-microbe interactions.

### 4.1. *In Vitro* Models to Study Intestinal Host-Microbe Interaction

One of the most straight-forward and widely used *in vitro* cell culture models to study host-microbe interactions are coculture systems. Typically, an intestinal epithelial cell line (e.g., Caco-2, HIECs, T84, IEC6, and HT29s) will be seeded as a monolayer, on transwells, or on a scaffold device. Bacteria, or bacterial supernatant, or other microbes, may be added to the culture chamber either directly to the cells or separated by some type of membrane or barrier [129–131]. Metabolic, molecular, and physiological assays can then be conducted in the hours or days following. These coculture experiments are scalable, highly reproducible, and straightforward to conduct in most labs with standard cell culture equipment. Additional cell types, such as primary-derived macrophages or PBMCs [132–134], can be included in the coculture. Despite the ease of performing these coculture experiments, they harbor limitations with regard to mimicking *in vivo* physiological conditions. To address this limitation, researchers have recently developed interesting *in vitro* coculture microfluidic, scaffold, and three-dimensional (3D) systems [135–138]. For example, Chen et al. developed a tube culture system to coculture enterocyte-like Caco-2 cells, Goblet-like HT29-MTX cells, and H-InMyoFibs myofibroblast cell lines. The tube structure allows researchers to pass media and bacteria across cells, while also mimicking the oxygen and nutrient gradients present *in vivo* within the intestinal tract [137]. Nonetheless, many of the cell lines used are transformed and therefore may not always faithfully represent primary cells. Moreover, there exist no known cell lines for certain intestinal cell types such as Paneth cells [45].

### 4.2. Ex Vivo Models to Study Intestinal Host-Microbe Interaction

More recently, researchers have moved to the use of ex vivo three-dimensional (3D) primary enteroid and intestinal organoid models to evaluate epithelial-microbe interactions [38, 93, 139–141]. Intestinal tissue is isolated and single cells, crypts, or whole mucosa is extracted and grown in a collagen-rich matrix, such as Matrigel. Enteroids and organoids will grow into large 3D masses containing all mature cell types of the isolated tissue, which more accurately mimics *in vivo* physiology compared to *in vitro* models [142]. Enteroids refers to cultures consisting solely of intestinal epithelial tissue, whereas organoids are derived to contain multiple tissue types, such as epithelia, enteric nerves, myofibroblasts, and smooth muscle cells [143]. Enteroid cultures can be passaged indefinitely making them a viable alternative to immortalized cell lines. Of note, these structures can also be derived using induced pluripotent stem cells (iPSC cells) [144, 145]. Because enteroids will form sealed “lumens,” with villi forming on the inside and crypts projecting outward, microbes should be microinjected into the lumens to evaluate host-microbe interactions (see [146, 147] for review). Microinjections of enteroids and organoids can be challenging. Moreover, the tendency of these structures to occasionally burst and then reseal can be prohibitive to long-term studies of injected microbes. Nevertheless, we recently demonstrated that IESCs grown in enteroid culture can be genetically manipulated using gymnosis to knockdown gene and microRNA expression [38]. Recently, monolayer versions of ex vivo enteroid culture systems have emerged, which expand the number of assays that can be performed, including patch clamps and live imaging studies [134, 148]. Less widely used are ex vivo mucosal explants and slice models, which, like organoids, contain a full complement of intestinal cell types [149, 150] and like coculture systems can be manipulated by adding microbes to the culture media (see review [151]). However, even with high oxygenation, small bowel explants have not been cultured successfully beyond 48 hours, and are not easily multiplexed like some enteroid systems [152], which severely limits their usefulness [149, 153]. Despite the advantages of using these culture systems, results of experiments intended to evaluate the effects on IESCs could be confounded by the presence of mature, differentiated intestinal cell types. Certain small molecules may assist in enriching for IESCs, for example, valproic acid and CHIR99021 [154], which could help clarify direct effects of microbes on IESCs.

### 4.3. *In Vivo* Models to Study Intestinal Host-Microbe Interaction

Finally, there are a number of *in vivo* methods to study the effect of microbiota on the intestinal stem cell niche. These models typically fall into one of two classes: introduction-based or depletion-based. In introduction models, a GF animal is exposed to microbes in a process termed “colonization.” Depletion models on the other hand aim to remove microbiota from a CR animal through the exposure to broad-spectrum antibiotics. Sometimes, researchers may combine approaches and reintroduce microbiota following depletion [175–177]. There are benefits and limitations to both approaches.

While the systemic and intestinal physiology of GF mice is atypical, these animals provide a “blank slate” for researchers to evaluate the effects of single strains, defined sets of microbes, or undefined microbiota on the stem cell niche. However, as humans are never reared in GF conditions, the clinical utility of GF models is often questioned [178]. Nevertheless, GF animals provide a valuable resource. Attempts at generating GF animals began before the beginning of the 20th century using chickens and guinea pigs [179, 180]. However, multigenerational GF animals were not described until much later in the 20th century (see [180, 181] for review). Currently, GF animals are acquired surgically through aseptic caesarian section or embryo transfer, and then maintained under sterile conditions in specialized isolation chambers. Food, water, and bedding must be sterilized prior to being introduced to animals, and fecal matter as well as cage environments are regularly checked to verify that no microbes have unintentionally been introduced. While GF animals survive, and in fact may live longer than CR animals [182], they develop abnormally and have altered behavior, metabolism, digestion, and immune system function [180]. Colonization of GF animals with microbes elicits a robust immune response, which takes several weeks to normalize to a state more similar to that of CR animals [166–168, 183]. The dynamic process of conventionalization is an important consideration as animal age, length of colonization, and animal diet contribute to microbial community structure and immune response. Moreover, colonization dynamics demonstrate substantial regional specificity. Temporal and regional dynamics of GF mouse conventionalization have been examined, most notably in a series of papers by El Aidy and colleagues [166–168, 184]. From these studies and others, we know certain developmental processes have a limited timeframe during which microbial colonization of GF animals may restore phenotypic similarity, especially within the immune system, with CR animals (see review [185]). Temporal and regional changes are also quite robust, with genes involved in innate immunity being most different in the first couple of days following colonization and stabilizing between 2 and 3 weeks postcolonization [167, 186, 187]. Regionally, immune cell recruitment occurs more rapidly in the small intestine compared to the colon in the days postcolonization [167], which has the potential to affect niche response. Many studies have performed colonization at different ages and for different lengths of time, making cross-study comparisons challenging. Moreover, differences in housing conditions, bedding material, and nonsterilized foods can introduce variables that further confound cross-study comparisons. The evaluation of the *in vivo* effect of specific microbes can be achieved using GF animals. However, because early microbe exposure significantly affects immune development and other physiological functions, the results of some gnotobiotic experiments may not reflect what occurs in animals that have been exposed to microbes since birth [188]. Despite these limitations, GF models have been used successfully to evaluate the effect of microbiota on IESCs, including many studies employing laser capture microdissection (LCM) [42, 85, 172, 189, 190] to isolate and test the effect of microbiota on the niche. For example, using LCM, Yu et al. [85] assessed the effects of microbiota on crypt cell gene expression following colonization of GF animals with microbiota collected from neonatal patient samples. Others have shown specific effects of antibiotics and colonization on gene expression in the intestinal crypts [172]. LCM of intestinal epithelial crypts includes several cell lineages, though it is possible to enrich for IESCs by genetically depleting Paneth cells [190]. This method, however, is labor intensive and does not result in high yields of RNA. As an alternative to LCM methods, we derived GF Sox9-EGFP reporter mice, which allow for the isolation of IESCs and progenitor cells using fluorescent-activated cell sorting (FACS), allowing for more precise assaying of cell-type-specific effects of microbiota [38].

Depletion of bacteria in CR animals using broad spectrum antibiotics is another approach for investigating the effect of microbiota on the stem cell niche. The major advantages are that such depletion-based approaches are substantially less expensive and quicker to conduct. However, there are several limitations. Notably, it has been shown that antibiotic treatment alone, irrespective of microbial depletion, can modify host gene expression and cause alterations to the intestinal epithelium, especially within the crypt compartment [172]. Moreover, complete elimination of microbiota using antibiotics is unlikely [169, 191], especially since most broad spectrum antibiotics specifically target bacteria, leaving enteric fungi and viruses to flourish. Nevertheless, antibiotic treatment continues to be a widely used model to investigate the effect of microbes on the host. It is likely that a combination of both introduction and depletion models could be helpful to evaluate fully the effect of microbial factors on the niche [192].

Another strategy that circumvents both gnotobiotic and antibiotic models is surgery to create isolated intestinal segments, such as Thiry-Vella fistulas, to determine the effect of autonomous microbial changes on intestinal function without experimental modification of the lumen [193]. However, this *in vivo* model eliminates normal luminal flow which of course does not properly reflect normal physiology. Despite the inherent limitations of all of the investigative methods, much has been learned concerning the mechanisms mediating microbial influences on host intestinal epithelial structure and function.

## 5. Conclusion and Discussion

The IESC niche constitutes a complex network of cell types expanding well beyond the epithelial layer to help govern the balance between IESC self-renewal and differentiation. The mammalian IESC is comprised of epithelial cells including IESCs, Paneth cells, and EECs, as well as nonepithelial components including stromal, neural, and immune cell types. It is also evident that gut microbiota have a prominent influence on intestinal epithelial physiology and stem cell function. However, the underlying mechanisms remain poorly understood and are still under active investigation. A major challenge is the isolation of functionally distinct cellular subpopulations and niche cells from the intestine as well as the difficulty in ascertaining the specific effect of individual microbes, metabolites, and other microbe-derived products. Several *in vitro*, ex vivo, and *in vivo* tools are available to investigate the relationship between host and microbe within the gut, and the research community has made substantial strides in the last decade. Nevertheless, several key questions remain, most notably the following: (1) Do IESCs respond to direct signals from gut microbiota? (2) Which niche cells are essential for proper microbial control of IESCs? (3) Do IESCs provide feedback to intestinal microbiota? (4) Does the niche contribute to the selection of microbes which reside in crypts, and what if any are the unique functions of the crypt-based microbes in regulating IESC behavior? (5) How are host-microbe interactions altered by diet, age, disease, or anatomic position along the GI tract? The answers to these questions will significantly advance our understanding of the role of host-microbe communication in normal intestinal physiology and in driving gastrointestinal diseases.

As we continue to address these and related important questions, moving forward, it is our opinion that special care must be taken to standardize relevant *in vitro*, ex vivo, and *in vivo* experiments in order to facilitate cross-study comparisons. For example, in terms of *in vivo* studies, given what we know of regional specificity and variability, we believe it is important whenever possible to report measurements from all three major small intestinal segments as well as the colon. Also, as rodents ingest bedding material, a considerable source of fiber, studies using animal models should include specifics as to bedding material, the diets used throughout the study course, the housing conditions (single versus cohoused, open versus closed ventilation, and light/dark cycles), the age at (and duration of) colonization, and the source, composition, and handling of the microbiota used for colonization, all of which have previously been shown to affect microbial composition.

The development of probiotics or engineered bacteria, as well as molecular strategies such as those based on microRNAs, represent exciting possibilities for modulating the gut microbiome and the IESC stem cell niche and thereby modifying intestinal physiology. Such efforts could in the long-term provide benefit to patients with a wide range of gastrointestinal diseases. With many recent advances in tools and technologies for exploring direct and indirect interactions between microbes and host IESCs, we anticipate significant progress in this area over the next decade.

## Figures and Tables

**Table 1:** 

Coculture type	Description	Pros	Cons	Reference
Monolayer	An intestinal cell line (or ex vivo enteroids) is grown in monolayers on standard cell culture plate or transwell. Bacteria are added to the media and cocultured for hours or days.	(i) Assay effects of single bacterial strain or pathogen on IECs (ii) Quick growth (iii) Good reproducibility (iv) Bacteria exposure remains apical (v) Easily multiplexed (vi) Coculture IECs with other intestinal niche cell types available (vii) Easy genetic manipulated in culture via transfections or infection (viii) Certain assays are more easily applied to monolayers	(i) Cell lines are somewhat homogenous and poorly reflect niche cell behavior (ii) Poorly reflect the regional specificity of the intestine (iii) Bacteria can quickly outgrow epithelial cells (iv) Monolayers poorly reflect IE conditions or mucus layer physiology (v) Certain niche cells lack representative cell lines	[129–134, 148, 155]

3D-scaffold	Intestinal cell lines (or ex vivo enteroids) are seeded onto a fabricated 3D-scaffold. Bacteria are added to the media and cocultured for hours, days, or months.	(i) Assay effects of single bacterial strain or pathogen on IECs (ii) Coculture IECs with other intestinal niche cell types (iii) Quick growth (iv) Depending on model, can better replicate movement, morphology, rigidity, oxygen, and nutrient gradients relative to monolayer models	(i) Difficult setup and/or specialized materials or parts (ii) May be difficult to multiplex	[135–138, 156–159]

Mucosal explant	Intestinal tissue biopsies or slices are taken, and mucosa/submucosa can be isolated and plated on cell culture plates or transwell inserts. Selected bacterial strains are added to the media and cocultured for hours or days.	(i) Can easily assay effects of single bacterial strain or community on primary tissue (ii) Better replicates *in vivo* environment than monolayer, cell line cultures (iii) Good viability in presence of commensal microbes (iv) Produce the wide range of metabolites and cytokines found *in vivo*	(i) Cannot be passaged or replicated (ii) May require specialized media and expensive growth factors (iii) Difficult to identify cell-type-specific effects/responses to microbiota	[149, 150, 160, 161]

Enteroids/organoids	IESCs or crypts are isolated fresh or derived from induced pluripotent or embryonic stem cells and suspended in a collagen-rich matrix (Matrigel). Growth factors are added to the media to support their growth. Bacteria should be injected into the lumen or added to the media, as enteroids/organoids form with the villi on the inside and crypts projecting outward.	(i) Assay effects of single bacterial strain or community on primary tissue (ii) Better replicates *in vivo* environment than monolayer, cell line cultures (iii) Produce the wide range of metabolites and cytokines found *in vivo* (iv) Can be passaged indefinitely and cryopreserved (v) Can easily be genetically manipulated in culture via transfections, gymnosis, or infection (vi) Can generate from patient-derived tissue or any available genetic model	(i) Injection of bacteria requires specialized equipment and expert technical skill (ii) May require specialized media and expensive growth factors	[38, 61, 139, 152, 162–165]

Introduction model	Animals are derived or maintained in a GF (gnotobiotic) facility. Selected bacterial strains or mixed microbiota (such as reconstituted fecal matter) are introduced to the animals.	(i) Can assay effects of mono- or polycolonization (ii) Can colonize with patient-derived microbiota (iii) Variables can be tightly controlled (iv) GF animals can be maintained under GF conditions indefinitely (v) Highly reproducible	(i) GF mice have altered development and physiology (ii) Limited number of genetic models readily available at most gnotobiotic facilities (iii) Expensive to generate and house	[85, 166–171]

Depletion model	CR or specific-pathogen free animals are given broad spectrum antibiotics, typically in drinking water, to remove measurable traces of microbiota. Microbiota may be reintroduced to the animals passively, or through forced colonization.	(i) Assay effects of single bacterial strain or community on primary tissue (ii) No need for gnotobiotic facility or equipment (iii) Very affordable to conduct (iv) More realistic in terms of human disease and physiology (v) Any available genetic model can be used	(i) Antibiotic treatment alters host gene expression independent of microbiota [172] (ii) Does not fully eliminate all microbes	[42, 91, 172–174]

## References

[1] van der Wath R. C., Gardiner B. S., Burgess A. W., Smith D. W. (2013). Cell organisation in the colonic crypt: a theoretical comparison of the pedigree and niche concepts. *PLoS One*.

[2] Barker N., van de Wetering M., Clevers H. C. (2008). The intestinal stem cell. *Genes & Development*.

[3] Clevers H. C. (2013). The intestinal crypt, a prototype stem cell compartment. *Cell*.

[4] Scadden D. T. (2006). The stem-cell niche as an entity of action. *Nature*.

[5] Pédron T., Mulet C., Dauga C. (2012). A crypt-specific core microbiota resides in the mouse colon. *MBio*.

[6] Verma S. C., Miyashiro T. (2016). Niche-specific impact of a symbiotic function on the persistence of microbial symbionts within a natural host. *Applied and Environmental Microbiology*.

[7] Biswas S., Davis H., Irshad S., Sandberg T., Worthley D., Leedham S. (2015). Microenvironmental control of stem cell fate in intestinal homeostasis and disease. *The Journal of Pathology*.

[8] Cramer J. M., Thompson T., Geskin A., LaFramboise W., Lagasse E. (2015). Distinct human stem cell populations in small and large intestine. *PLoS One*.

[9] Nigro G., Sansonetti P. J. (2015). Microbiota and gut stem cells cross-talks: a new view of epithelial homeostasis. *Current Stem Cell Reports*.

[10] Sommer F., Bäckhed F. (2013). The gut microbiota—masters of host development and physiology. *Nature Reviews Microbiology*.

[11] Tan D. W.-M., Barker N. (2014). Intestinal stem cells and their defining niche. *Current Topics in Developmental Biology*.

[12] De Mey J. R., Freund J.-N. (2013). Understanding epithelial homeostasis in the intestine: an old battlefield of ideas, recent breakthroughs and remaining controversies. *Tissue Barriers*.

[13] Simons B. D., Clevers H. C. (2011). Strategies for homeostatic stem cell self-renewal in adult tissues. *Cell*.

[14] van der Flier L. G., Clevers H. C. (2009). Stem cells, self-renewal, and differentiation in the intestinal epithelium. *Annual Review of Physiology*.

[15] Grossmann J., Walther K., Artinger M., Kiessling S., Schölmerich J. (2001). Apoptotic signaling during initiation of detachment-induced apoptosis (“anoikis”) of primary human intestinal epithelial cells. *Cell Growth & Differentiation*.

[16] Dufour G., Demers M.-J., Gagné D. (2004). Human intestinal epithelial cell survival and anoikis. Differentiation state-distinct regulation and roles of protein kinase B/Akt isoforms. *The Journal of Biological Chemistry*.

[17] Garabedian E. M., Roberts L. J., McNevin M. S., Gordon J. I. (1997). Examining the role of Paneth cells in the small intestine by lineage ablation in transgenic mice. *The Journal of Biological Chemistry*.

[18] Barker N. (2013). Adult intestinal stem cells: critical drivers of epithelial homeostasis and regeneration. *Nature Reviews Immunology*.

[19] Bohórquez D. V., Shahid R. A., Erdmann A. (2015). Neuroepithelial circuit formed by innervation of sensory enteroendocrine cells. *Journal of Clinical Investigation*.

[20] Tsubouchi S., Leblond C. P. (1979). Migration and turnover of entero-endocrine and caveolated cells in the epithelium of the descending colon, as shown by radioautography after continuous infusion of 3H-thymidine into mice. *The American Journal of Anatomy*.

[21] Potten C. S., Morris R. J. (1988). Epithelial stem cells in vivo. *Journal of Cell Science*.

[22] Moossavi S. (2014). Heterogeneity of the level of activity of lgr5+ intestinal stem cells. *International Journal of Molecular and Cellular Medicine*.

[23] Nair G., Abranches E., Guedes A. M. V., Henrique D., Raj A. (2015). Heterogeneous lineage marker expression in naive embryonic stem cells is mostly due to spontaneous differentiation. *Scientific Reports*.

[24] Muñoz J., Stange D. E., Schepers A. G. (2012). The Lgr5 intestinal stem cell signature: robust expression of proposed quiescent “+4” cell markers. *The EMBO Journal*.

[25] Roche K. C., Gracz A. D., Liu X. F., Newton V., Akiyama H., Magness S. T. (2015). SOX9 maintains reserve stem cells and preserves radioresistance in mouse small intestine. *Gastroenterology*.

[26] van Es J. H., Sato T., van de Wetering M. (2012). Dll1+ secretory progenitor cells revert to stem cells upon crypt damage. *Nature Cell Biology*.

[27] Tetteh P. W., Basak O., Farin H. F. (2016). Replacement of lost Lgr5-positive stem cells through plasticity of their enterocyte-lineage daughters. *Cell Stem Cell*.

[28] Sipos F., Műzes G. (2015). Injury-associated reacquiring of intestinal stem cell function. *World Journal of Gastroenterology*.

[29] Jadhav U., Saxena M., O’Neill N. K. (2017). Dynamic reorganization of chromatin accessibility signatures during dedifferentiation of secretory precursors into Lgr5+ intestinal stem cells. *Cell Stem Cell*.

[30] Smith R. J., Rao-Bhatia A., Kim T.-H. (2017). Signaling and epigenetic mechanisms of intestinal stem cells and progenitors: insight into crypt homeostasis, plasticity, and niches. *Wiley Interdisciplinary Reviews: Developmental Biology*.

[31] Beumer J., Clevers H. C. (2016). Regulation and plasticity of intestinal stem cells during homeostasis and regeneration. *Development*.

[32] Grün D., Lyubimova A., Kester L. (2015). Single-cell messenger RNA sequencing reveals rare intestinal cell types. *Nature*.

[33] von Furstenberg R. J., Gulati A. S., Baxi A. (2011). Sorting mouse jejunal epithelial cells with CD24 yields a population with characteristics of intestinal stem cells. *American Journal of Physiology Gastrointestinal and Liver Physiology*.

[34] Yan K. S., Gevaert O., Zheng G. X. Y. (2017). Intestinal enteroendocrine lineage cells possess homeostatic and injury-inducible stem cell activity. *Cell Stem Cell*.

[35] Mah A. T., Kuo C. J. (2016). Home sweet home: a Foxl1(+) mesenchymal niche for intestinal stem cells. *Cellular and Molecular Gastroenterology and Hepatology*.

[36] Walker E. M., Thompson C. A., Battle M. A. (2014). GATA4 and GATA6 regulate intestinal epithelial cytodifferentiation during development. *Developmental Biology*.

[37] Peck B. C. E., Sincavage J., Feinstein S. (2016). Mir-30 family controls proliferation and differentiation of intestinal epithelial cell models by directing a broad gene expression program that includes SOX9 and the ubiquitin ligase pathway. *The Journal of Biological Chemistry*.

[38] Peck B. C. E., Mah A. T., Pitman W. A., Ding S., Lund P. K., Sethupathy P. (2017). Functional transcriptomics in diverse intestinal epithelial cell types reveals robust microRNA sensitivity in intestinal stem cells to microbial status. *The Journal of Biological Chemistry*.

[39] Henning S. J., von Furstenberg R. J. (2016). GI stem cells - new insights into roles in physiology and pathophysiology. *The Journal of Physiology*.

[40] Clevers H. C., Bevins C. L. (2013). Paneth cells: maestros of the small intestinal crypts. *Annual Review of Physiology*.

[41] Durand A., Donahue B., Peignon G. (2012). Functional intestinal stem cells after Paneth cell ablation induced by the loss of transcription factor Math1 (Atoh1). *Proceedings of the National Academy of Sciences of the United States of America*.

[42] Vaishnava S., Behrendt C. L., Ismail A. S., Eckmann L., Hooper L. V. (2008). Paneth cells directly sense gut commensals and maintain homeostasis at the intestinal host-microbial interface. *Proceedings of the National Academy of Sciences of the United States of America*.

[43] Sasaki N., Sachs N., Wiebrands K. (2016). Reg4+ deep crypt secretory cells function as epithelial niche for Lgr5+ stem cells in colon. *Proceedings of the National Academy of Sciences of the United States of America*.

[44] Ouellette A. J. (2010). Paneth cells and innate mucosal immunity. *Current Opinion in Gastroenterology*.

[45] Porter E. M., Bevins C. L., Ghosh D., Ganz T. (2002). The multifaceted Paneth cell. *Cellular and Molecular Life Sciences*.

[46] Gunawardene A. R., Corfe B. M., Staton C. A. (2011). Classification and functions of enteroendocrine cells of the lower gastrointestinal tract. *International Journal of Experimental Pathology*.

[47] Cani P. D., Everard A., Duparc T. (2013). Gut microbiota, enteroendocrine functions and metabolism. *Current Opinion in Pharmacology*.

[48] Sykaras A. G., Demenis C., Cheng L. (2014). Duodenal CCK cells from male mice express multiple hormones including ghrelin. *Endocrinology*.

[49] Radford I. R., Lobachevsky P. N. (2006). An enteroendocrine cell-based model for a quiescent intestinal stem cell niche. *Cell Proliferation*.

[50] Furness J. B., Rivera L. R., Cho H.-J., Bravo D. M., Callaghan B. (2013). The gut as a sensory organ. *Nature Reviews Gastroenterology & Hepatology*.

[51] Aiken K. D., Kisslinger J. A., Roth K. A. (1994). Immunohistochemical studies indicate multiple enteroendocrine cell differentiation pathways in the mouse proximal small intestine. *Developmental Dynamics*.

[52] Shamsuddin A. M., Phelps P. C., Trump B. F. (1982). Human large intestinal epithelium: light microscopy, histochemistry, and ultrastructure. *Human Pathology*.

[53] Sjölund K., Sandén G., Håkanson R., Sundler F. (1983). Endocrine cells in human intestine: an immunocytochemical study. *Gastroenterology*.

[54] Cristina M. L., Lehy T., Zeitoun P., Dufougeray F. (1978). Fine structural classification and comparative distribution of endocrine cells in normal human large intestine. *Gastroenterology*.

[55] Buczacki S. J. A., Zecchini H. I., Nicholson A. M. (2013). Intestinal label-retaining cells are secretory precursors expressing Lgr5. *Nature*.

[56] Li N., Nakauka-Ddamba A., Tobias J., Jensen S. T., Lengner C. J. (2016). Mouse label-retaining cells are molecularly and functionally distinct from reserve intestinal stem cells. *Gastroenterology*.

[57] Amato A., Baldassano S., Mulè F. (2016). GLP2: an underestimated signal for improving glycaemic control and insulin sensitivity. *The Journal of Endocrinology*.

[58] Mazzoli R., Pessione E. (2016). The neuro-endocrinological role of microbial glutamate and GABA signaling. *Frontiers in Microbiology*.

[59] Breton J., Tennoune N., Lucas N. (2016). Gut commensal E. coli proteins activate host satiety pathways following nutrient-induced bacterial growth. *Cell Metabolism*.

[60] Le Guen L., Marchal S., Faure S., de Santa Barbara P. (2015). Mesenchymal-epithelial interactions during digestive tract development and epithelial stem cell regeneration. *Cellular and Molecular Life Sciences*.

[61] Stzepourginski I., Nigro G., Jacob J.-M. (2017). CD34+ mesenchymal cells are a major component of the intestinal stem cells niche at homeostasis and after injury. *Proceedings of the National Academy of Sciences of the United States of America*.

[62] Aoki R., Shoshkes-Carmel M., Gao N. (2016). Foxl1-expressing mesenchymal cells constitute the intestinal stem cell niche. *Cellular and Molecular Gastroenterology and Hepatology*.

[63] Bain C. C., Mowat A. M. (2014). Macrophages in intestinal homeostasis and inflammation. *Immunological Reviews*.

[64] McDole J. R., Wheeler L. W., McDonald K. G. (2012). Goblet cells deliver luminal antigen to CD103^+^ dendritic cells in the small intestine. *Nature*.

[65] Gerbe F., Sidot E., Smyth D. J. (2016). Intestinal epithelial tuft cells initiate type 2 mucosal immunity to helminth parasites. *Nature*.

[66] Gerbe F., Jay P. (2016). Intestinal tuft cells: epithelial sentinels linking luminal cues to the immune system. *Mucosal Immunology*.

[67] von Moltke J., Ji M., Liang H. E., Locksley R. M. (2016). Tuft-cell-derived IL-25 regulates an intestinal ILC2-epithelial response circuit. *Nature*.

[68] Gronke K., Diefenbach A. (2016). Tuft cell-derived IL-25 activates and maintains ILC2. *Immunology and Cell Biology*.

[69] Saha S., Aranda E., Hayakawa Y. (2016). Macrophage-derived extracellular vesicle-packaged WNTs rescue intestinal stem cells and enhance survival after radiation injury. *Nature Communications*.

[70] Lundgren O., Jodal M., Jansson M., Ryberg A. T., Svensson L. (2011). Intestinal epithelial stem/progenitor cells are controlled by mucosal afferent nerves. *PLoS One*.

[71] Lasrado R., Boesmans W., Kleinjung J. (2017). Lineage-dependent spatial and functional organization of the mammalian enteric nervous system. *Science*.

[72] Van Landeghem L., Chevalier J., Mahe M. M. (2011). Enteric glia promote intestinal mucosal healing via activation of focal adhesion kinase and release of proEGF. *American Journal of Physiology Gastrointestinal and Liver Physiology*.

[73] Neunlist M., Rolli-Derkinderen M., Latorre R. (2014). Enteric glial cells: recent developments and future directions. *Gastroenterology*.

[74] Sailaja B. S., He X. C., Li L. (2016). The regulatory niche of intestinal stem cells. *The Journal of Physiology*.

[75] Yen T.-H., Wright N. A. (2006). The gastrointestinal tract stem cell niche. *Stem Cell Reviews*.

[76] Gordon H. A., Bruckner-Kardoss E. (1961). Effects of the normal microbial flora on various tissue elements of the small intestine. *Cells, Tissues, Organs*.

[77] Abrams G. D. (1977). Microbial effects on mucosal structure and function. *American Journal of Clinical Nutrition*.

[78] Lesher S., Walburg H. E., Sacher G. A. (1964). Generation cycle in the duodenal crypt cells of germ-free and conventional mice. *Nature*.

[79] Clarke R. M. (1975). Diet, mucosal architecture and epithelial cell production in the small intestine of specified-pathogen-free and conventional rats. *Laboratory Animals*.

[80] Smith K., McCoy K. D., Macpherson A. J. (2007). Use of axenic animals in studying the adaptation of mammals to their commensal intestinal microbiota. *Seminars in Immunology*.

[81] Satoh Y., Ishikawa K., Tanaka H., Ono K. (1986). Immunohistochemical observations of immunoglobulin A in the Paneth cells of germ-free and formerly-germ-free rats. *Histochemistry*.

[82] Khoury K. A., Floch M. H., Hersh T. (1969). Small intestinal mucosal cell proliferation and bacterial flora in the conventionalization of the germfree mouse. *The Journal of Experimental Medicine*.

[83] Thompson G. R., Trexler P. C. (1971). Gastrointestinal structure and function in germ-free or gnotobiotic animals. *Gut*.

[84] Khoury K. A., Floch M. H., Herskovic T. (1969). Effects of neomycin and penicillin administration on mucosal proliferation of the mouse small intestine. With morphological and functional correlations. *The Journal of Experimental Medicine*.

[85] Yu Y., Lu L., Sun J., Petrof E. O., Claud E. C. (2016). Preterm infant gut microbiota affects intestinal epithelial development in a humanized microbiome gnotobiotic mouse model. *American Journal of Physiology Gastrointestinal and Liver Physiology*.

[86] Alam M., Midtvedt T., Uribe A. (1994). Differential cell kinetics in the ileum and colon of germfree rats. *Scandinavian Journal of Gastroenterology*.

[87] Nowacki M. R. (1993). Cell proliferation in colonic crypts of germ-free and conventional mice—preliminary report. *Folia Histochemica et Cytobiologica*.

[88] Meslin J. C., Sacquet E. (1984). Effects of microflora on the dimensions of enterocyte microvilli in the rat. *Reproduction, Nutrition, Development*.

[89] Riottot M., Sacquet E. (1985). Increase in the ileal absorption rate of sodium taurocholate in germ-free or conventional rats given an amylomaize-starch diet. *The British Journal of Nutrition*.

[90] Darwich A. S., Aslam U., Ashcroft D. M., Rostami-Hodjegan A. (2014). Meta-analysis of the turnover of intestinal epithelia in preclinical animal species and humans. *Drug Metabolism and Disposition*.

[91] Preidis G. A., Saulnier D. M., Blutt S. E. (2012). Probiotics stimulate enterocyte migration and microbial diversity in the neonatal mouse intestine. *FASEB Journal*.

[92] Kernbauer E., Ding Y., Cadwell K. (2014). An enteric virus can replace the beneficial function of commensal bacteria. *Nature*.

[93] Gong W., Guo M., Han Z. (2016). Mesenchymal stem cells stimulate intestinal stem cells to repair radiation-induced intestinal injury. *Cell Death & Disease*.

[94] Johansson M. E. V., Phillipson M., Petersson J., Velcich A., Holm L., Hansson G. C. (2008). The inner of the two Muc2 mucin-dependent mucus layers in colon is devoid of bacteria. *Proceedings of the National Academy of Sciences of the United States of America*.

[95] Hansson G. C., Johansson M. E. (2010). The inner of the two Muc2 mucin-dependent mucus layers in colon is devoid of bacteria. *Gut Microbes*.

[96] Johansson M. E. V., Gustafsson J. K., Holmén-Larsson J. (2013). Bacteria penetrate the normally impenetrable inner colon mucus layer in both murine colitis models and patients with ulcerative colitis. *Gut*.

[97] Savage D. C., Blumershine R. V. (1974). Surface-surface associations in microbial communities populating epithelial habitats in the murine gastrointestinal ecosystem: scanning electron microscopy. *Infection and Immunity*.

[98] Palestrant D., Holzknecht Z. E., Collins B. H., Parker W., Miller S. E., Bollinger R. R. (2004). Microbial biofilms in the gut: visualization by electron microscopy and by acridine orange staining. *Ultrastructural Pathology*.

[99] Donaldson G. P., Lee S. M., Mazmanian S. K. (2016). Gut biogeography of the bacterial microbiota. *Nature Reviews Immunology*.

[100] Nelson D. P., Mata L. J. (1970). Bacterial flora associated with the human gastrointestinal mucosa. *Gastroenterology*.

[101] Swidsinski A., Weber J., Loening-Baucke V., Hale L.-P., Lochs H. (2005). Spatial organization and composition of the mucosal flora in patients with inflammatory bowel disease. *Journal of Clinical Microbiology*.

[102] Swidsinski A., Loening-Baucke V., Lochs H., Hale L.-P. (2005). Spatial organization of bacterial flora in normal and inflamed intestine: a fluorescence in situ hybridization study in mice. *World Journal of Gastroenterology*.

[103] Earle K. A., Billings G., Sigal M. (2015). Quantitative imaging of gut microbiota spatial organization. *Cell Host & Microbe*.

[104] Corrodi P., Wideman P. A., Sutter V. L., Drenick E. J., Passaro E., Finegold S. M. (1978). Bacterial flora of the small bowel before and after bypass procedure for morbid obesity. *The Journal of Infectious Diseases*.

[105] Hayashi H., Takahashi R., Nishi T., Sakamoto M., Benno Y. (2005). Molecular analysis of jejunal, ileal, caecal and recto-sigmoidal human colonic microbiota using 16S rRNA gene libraries and terminal restriction fragment length polymorphism. *Journal of Medical Microbiology*.

[106] Keilberg D., Zavros Y., Shepherd B., Salama N. R., Ottemann K. M. (2016). Spatial and temporal shifts in bacterial biogeography and gland occupation during the development of a chronic infection. *MBio*.

[107] Vanaja S. K., Russo A. J., Behl B. (2016). Bacterial outer membrane vesicles mediate cytosolic localization of LPS and caspase-11 activation. *Cell*.

[108] Tyrer P. C., Frizelle F. A., Keenan J. I. (2014). *Escherichia coli*-derived outer membrane vesicles are genotoxic to human enterocyte-like cells. *Infectious Agents and Cancer*.

[109] Shen Y., Giardino Torchia M. L., Lawson G. W., Karp C. L., Ashwell J. D., Mazmanian S. K. (2012). Outer membrane vesicles of a human commensal mediate immune regulation and disease protection. *Cell Host & Microbe*.

[110] O'Donoghue E. J., Krachler A. M. (2016). Mechanisms of outer membrane vesicle entry into host cells. *Cellular Microbiology*.

[111] Kunsmann L., Rüter C., Bauwens A. (2015). Virulence from vesicles: novel mechanisms of host cell injury by Escherichia coli O104:H4 outbreak strain. *Scientific Reports*.

[112] Kaiko G. E., Ryu S. H., Koues O. I. (2016). The colonic crypt protects stem cells from microbiota-derived metabolites. *Cell*.

[113] Lee W.-J., Hase K. (2014). Gut microbiota-generated metabolites in animal health and disease. *Nature Chemical Biology*.

[114] Kaiko G. E., Ryu S. H., Koues O. I. (2016). The colonic crypt protects stem cells from microbiota-derived metabolites. *Cell*.

[115] Magnúsdóttir S., Ravcheev D., de Crécy-Lagard V., Thiele I. (2015). Systematic genome assessment of B-vitamin biosynthesis suggests co-operation among gut microbes. *Frontiers in Genetics*.

[116] Bevins C. L., Salzman N. H. (2011). Paneth cells, antimicrobial peptides and maintenance of intestinal homeostasis. *Nature Reviews Microbiology*.

[117] Shanahan M. T., Carroll I. M., Gulati A. S. (2014). Critical design aspects involved in the study of Paneth cells and the intestinal microbiota. *Gut Microbes*.

[118] Ouellette A. J., Selsted M. E. (1996). Paneth cell defensins: endogenous peptide components of intestinal host defense. *FASEB Journal*.

[119] Larraufie P., Doré J., Lapaque N., Blottière H. M. (2017). TLR ligands and butyrate increase Pyy expression through two distinct but inter-regulated pathways. *Cellular Microbiology*.

[120] Rumio C., Besusso D., Palazzo M. (2004). Degranulation of Paneth cells via Toll-like receptor 9. *The American Journal of Pathology*.

[121] Rumio C., Sommariva M., Sfondrini L. (2012). Induction of Paneth cell degranulation by orally administered Toll-like receptor ligands. *Journal of Cellular Physiology*.

[122] Giorgetti G., Brandimarte G., Fabiocchi F. (2015). Interactions between innate immunity, microbiota, and probiotics. *Journal of Immunology Research*.

[123] Mannon P. J. (2002). Peptide YY as a growth factor for intestinal epithelium. *Peptides*.

[124] Aponte G. W. (2002). PYY-mediated fatty acid induced intestinal differentiation. *Peptides*.

[125] Howitt M. R., Lavoie S., Michaud M. (2016). Tuft cells, taste-chemosensory cells, orchestrate parasite type 2 immunity in the gut. *Science*.

[126] Humphreys N. E., Xu D., Hepworth M. R., Liew F. Y., Grencis R. K. (2008). IL-33, a potent inducer of adaptive immunity to intestinal nematodes. *The Journal of Immunology*.

[127] Mahapatro M., Foersch S., Hefele M. (2016). Programming of intestinal epithelial differentiation by IL-33 derived from pericryptal fibroblasts in response to systemic infection. *Cell Reports*.

[128] Neunlist M., Schemann M. (2014). Nutrient-induced changes in the phenotype and function of the enteric nervous system. *The Journal of Physiology*.

[129] Kim J. M., Jung H. C., Im K. I., Song I. S., Kim C. Y. (1998). Synergy between Entamoeba histolytica and Escherichia coli in the induction of cytokine gene expression in human colon epithelial cells. *Parasitology Research*.

[130] Furrie E., Macfarlane S., Thomson G., Macfarlane G. T., Microbiology & Gut Biology Group, Tayside Tissue & Tumour Bank (2005). Toll-like receptors-2, -3 and -4 expression patterns on human colon and their regulation by mucosal-associated bacteria. *Immunology*.

[131] Varon C., Duriez A., Lehours P. (2009). Study of Helicobacter pullorum proinflammatory properties on human epithelial cells in vitro. *Gut*.

[132] Haller D., Serrant P., Peruisseau G. (2002). IL-10 producing CD14^Low^ monocytes inhibit lymphocyte-dependent activation of intestinal epithelial cells by commensal bacteria. *Microbiology and Immunology*.

[133] Diebel L. N., Liberati D. M., Taub J. S., Diglio C. A., Brown W. J. (2005). Intestinal epithelial cells modulate PMN activation and apoptosis following bacterial and hypoxic challenges. *The Journal of Trauma*.

[134] Noel G., Baetz N. W., Staab J. F. (2017). A primary human macrophage-enteroid co-culture model to investigate mucosal gut physiology and host-pathogen interactions. *Scientific Reports*.

[135] Kim J., Hegde M., Jayaraman A. (2010). Microfluidic co-culture of epithelial cells and bacteria for investigating soluble signal-mediated interactions. *Journal of Visualized Experiments*.

[136] Kim J., Hegde M., Jayaraman A. (2010). Co-culture of epithelial cells and bacteria for investigating host–pathogen interactions. *Lab on a Chip*.

[137] Chen Y., Lin Y., Davis K. M. (2015). Robust bioengineered 3D functional human intestinal epithelium. *Scientific Reports*.

[138] Costello C. M., Sorna R. M., Goh Y.-L., Cengic I., Jain N. K., March J. C. (2014). 3-D intestinal scaffolds for evaluating the therapeutic potential of probiotics. *Molecular Pharmaceutics*.

[139] Sato T., Vries R. G., Snippert H. J. (2009). Single Lgr5 stem cells build crypt-villus structures in vitro without a mesenchymal niche. *Nature*.

[140] Mah A. T., Van Landeghem L., Gavin H. E., Magness S. T., Lund P. K. (2014). Impact of diet-induced obesity on intestinal stem cells: hyperproliferation but impaired intrinsic function that requires insulin/IGF1. *Endocrinology*.

[141] Saxena K., Simon L. M., Zeng X.-L. (2017). A paradox of transcriptional and functional innate interferon responses of human intestinal enteroids to enteric virus infection. *Proceedings of the National Academy of Sciences of the United States of America*.

[142] Zachos N. C., Kovbasnjuk O., Foulke-Abel J. (2016). Human enteroids/colonoids and intestinal organoids functionally recapitulate normal intestinal physiology and pathophysiology. *The Journal of Biological Chemistry*.

[143] Stelzner M., Helmrath M., Dunn J. C. Y. (2012). A nomenclature for intestinal in vitro cultures. *American Journal of Physiology Gastrointestinal and Liver Physiology*.

[144] Sinagoga K. L., Wells J. M. (2015). Generating human intestinal tissues from pluripotent stem cells to study development and disease. *The EMBO Journal*.

[145] McCracken K. W., Howell J. C., Wells J. M., Spence J. R. (2011). Generating human intestinal tissue from pluripotent stem cells in vitro. *Nature Protocols*.

[146] Foulke-Abel J., In J., Kovbasnjuk O. (2014). Human enteroids as an ex-vivo model of host-pathogen interactions in the gastrointestinal tract. *Experimental Biology and Medicine*.

[147] In J. G., Foulke-Abel J., Estes M. K., Zachos N. C., Kovbasnjuk O., Donowitz M. (2016). Human mini-guts: new insights into intestinal physiology and host-pathogen interactions. *Nature Reviews Gastroenterology & Hepatology*.

[148] Psichas A., Tolhurst G., Brighton C. A., Gribble F. M., Reimann F. (2017). Mixed primary cultures of murine small intestine intended for the study of gut hormone secretion and live cell imaging of enteroendocrine cells. *Journal of Visualized Experiments*.

[149] Schwerdtfeger L. A., Ryan E. P., Tobet S. A. (2016). An organotypic slice model for ex vivo study of neural, immune, and microbial interactions of mouse intestine. *American Journal of Physiology Gastrointestinal and Liver Physiology*.

[150] van de Kerkhof E. G., de Graaf I. A. M., de Jager M. H., Meijer D. K. F., Groothuis G. M. M. (2005). Characterization of rat small intestinal and colon precision-cut slices as an in vitro system for drug metabolism and induction studies. *Drug Metabolism and Disposition*.

[151] Groothuis G. M. M., de Graaf I. A. M. (2013). Precision-cut intestinal slices as in vitro tool for studies on drug metabolism. *Current Drug Metabolism*.

[152] Gracz A. D., Williamson I. A., Roche K. C. (2015). A high-throughput platform for stem cell niche co-cultures and downstream gene expression analysis. *Nature Cell Biology*.

[153] Randall K. J., Turton J., Foster J. R., Masters J. (2011). Explant culture of gastrointestinal tissue: developing a tool for toxicology. *Toxicology*.

[154] Yin X., Farin H. F., van Es J. H., Clevers H. C., Langer R., Karp J. M. (2013). Niche-independent high-purity cultures of Lgr5^+^ intestinal stem cells and their progeny. *Nature Methods*.

[155] Zoumpopoulou G., Tsakalidou E., Dewulf J., Pot B., Grangette C. (2009). Differential crosstalk between epithelial cells, dendritic cells and bacteria in a co-culture model. *International Journal of Food Microbiology*.

[156] Kim S. H., Chi M., Yi B. (2014). Three-dimensional intestinal villi epithelium enhances protection of human intestinal cells from bacterial infection by inducing mucin expression. *Integrative Biology*.

[157] Juuti-Uusitalo K., Klunder L. J., Sjollema K. A. (2011). Differential effects of TNF (TNFSF2) and IFN-γ on intestinal epithelial cell morphogenesis and barrier function in three-dimensional culture. *PLoS One*.

[158] De Weirdt R., Crabbé A., Roos S. (2012). Glycerol supplementation enhances *L. reuteri*’s protective effect against *S.* Typhimurium colonization in a 3-D model of colonic epithelium. *PLoS One*.

[159] Mappley L. J., Tchórzewska M. A., Cooley W. A., Woodward M. J., La Ragione R. M. (2011). Lactobacilli antagonize the growth, motility, and adherence of Brachyspira pilosicoli: a potential intervention against avian intestinal spirochetosis. *Applied and Environmental Microbiology*.

[160] Borruel N., Casellas F., Antolín M. (2003). Effects of nonpathogenic bacteria on cytokine secretion by human intestinal mucosa. *The American Journal of Gastroenterology*.

[161] Tixier E., Lalanne F., Just I., Galmiche J.-P., Neunlist M. (2005). Human mucosa/submucosa interactions during intestinal inflammation: involvement of the enteric nervous system in interleukin-8 secretion. *Cellular Microbiology*.

[162] Gracz A. D., Puthoff B. J., Magness S. T. (2012). Identification, isolation, and culture of intestinal epithelial stem cells from murine intestine. *Methods in Molecular Biology*.

[163] Lukovac S., Belzer C., Pellis L. (2014). Differential modulation by *Akkermansia muciniphila* and *Faecalibacterium prausnitzii* of host peripheral lipid metabolism and histone acetylation in mouse gut organoids. *MBio*.

[164] Wilson S. S., Tocchi A., Holly M. K., Parks W. C., Smith J. G. (2015). A small intestinal organoid model of non-invasive enteric pathogen-epithelial cell interactions. *Mucosal Immunology*.

[165] Zhang X. T., Gong A. Y., Wang Y. (2016). Cryptosporidium parvum infection attenuates the ex vivo propagation of murine intestinal enteroids. *Physiological Reports*.

[166] El Aidy S., Merrifield C. A., Derrien M. (2013). The gut microbiota elicits a profound metabolic reorientation in the mouse jejunal mucosa during conventionalisation. *Gut*.

[167] El Aidy S., van Baarlen P., Derrien M. (2012). Temporal and spatial interplay of microbiota and intestinal mucosa drive establishment of immune homeostasis in conventionalized mice. *Mucosal Immunology*.

[168] El Aidy S., Hooiveld G., Tremaroli V., Bäckhed F., Kleerebezem M. (2013). The gut microbiota and mucosal homeostasis: colonized at birth or at adulthood, does it matter?. *Gut Microbes*.

[169] Ellekilde M., Selfjord E., Larsen C. S. (2014). Transfer of gut microbiota from lean and obese mice to antibiotic-treated mice. *Scientific Reports*.

[170] Liu S., da Cunha A. P., Rezende R. M. (2016). The host shapes the gut microbiota via fecal microRNA. *Cell Host & Microbe*.

[171] Stappenbeck T. S., Hooper L. V., Gordon J. I. (2002). Developmental regulation of intestinal angiogenesis by indigenous microbes via Paneth cells. *Proceedings of the National Academy of Sciences of the United States of America*.

[172] Morgun A., Dzutsev A., Dong X. (2015). Uncovering effects of antibiotics on the host and microbiota using transkingdom gene networks. *Gut*.

[173] Hörmann N., Brandão I., Jäckel S. (2014). Gut microbial colonization orchestrates TLR2 expression, signaling and epithelial proliferation in the small intestinal mucosa. *PLoS One*.

[174] Gounder A. P., Myers N. D., Treuting P. M. (2016). Defensins potentiate a neutralizing antibody response to enteric viral infection. *PLoS Pathogens*.

[175] Möhle L., Mattei D., Heimesaat M. M. (2016). Ly6C^hi^ monocytes provide a link between antibiotic-induced changes in gut microbiota and adult hippocampal neurogenesis. *Cell Reports*.

[176] Gacias M., Gaspari S., Santos P., Tamburini S. (2016). Microbiota-driven transcriptional changes in prefrontal cortex override genetic differences in social behavior. *eLife*.

[177] Lichtman J. S., Ferreyra J. A., Ng K. M., Smits S. A., Sonnenburg J. L., Elias J. E. (2016). Host-microbiota interactions in the pathogenesis of antibiotic-associated diseases. *Cell Reports*.

[178] Arrieta M.-C., Walter J., Finlay B. B. (2016). Human microbiota-associated mice: a model with challenges. *Cell Host & Microbe*.

[179] Nuttall G. H. F., Thierfelder H. (1897). Thierisches leben ohne bakterien im verdauungskanal. *Zeitschrift für Physiologische Chemie*.

[180] Luczynski P., McVey Neufeld K.-A., Oriach C. S., Clarke G., Dinan T. G., Cryan J. F. (2016). Growing up in a bubble: using germ-free animals to assess the influence of the gut microbiota on brain and behavior. *International Journal of Neuropsychopharmacology*.

[181] Levenson S. M., Mason R. P., Huber T. E., Malm O. J., Horowitz R. E., Einheber A. (1959). Germfree animals in surgical research. *Annals of Surgery*.

[182] Reyniers J. A., Sacksteder M. R. Observations on the survival of germfree C3H mice and their resistance to a contaminated environment.

[183] Gillilland M. G., Erb-Downward J. R., Bassis C. M. (2012). Ecological succession of bacterial communities during conventionalization of germ-free mice. *Applied and Environmental Microbiology*.

[184] El Aidy S., Kleerebezem M. (2013). Molecular signatures for the dynamic process of establishing intestinal host-microbial homeostasis: potential for disease diagnostics?. *Current Opinion in Gastroenterology*.

[185] Gensollen T., Iyer S. S., Kasper D. L., Blumberg R. S. (2016). How colonization by microbiota in early life shapes the immune system. *Science*.

[186] Larsson E., Tremaroli V., Lee Y. S. (2012). Analysis of gut microbial regulation of host gene expression along the length of the gut and regulation of gut microbial ecology through MyD88. *Gut*.

[187] Hansen C. H. F., Nielsen D. S., Kverka M. (2012). Patterns of early gut colonization shape future immune responses of the host. *PLoS One*.

[188] Falk P. G., Hooper L. V., Midtvedt T., Gordon J. I. (1998). Creating and maintaining the gastrointestinal ecosystem: what we know and need to know from gnotobiology. *Microbiology and Molecular Biology Reviews*.

[189] Bäckhed F., Ding H., Wang T. (2004). The gut microbiota as an environmental factor that regulates fat storage. *Proceedings of the National Academy of Sciences of the United States of America*.

[190] Stappenbeck T. S., Mills J. C., Gordon J. I. (2003). Molecular features of adult mouse small intestinal epithelial progenitors. *Proceedings of the National Academy of Sciences of the United States of America*.

[191] Hansen A. K. (1995). Antibiotic treatment of nude rats and its impact on the aerobic bacterial flora. *Laboratory Animals*.

[192] Lundberg R., Toft M. F., August B., Hansen A. K., Hansen C. H. F. (2016). Antibiotic-treated versus germ-free rodents for microbiota transplantation studies. *Gut Microbes*.

[193] Kern S. E., Keren D. F., Pierson C. L. (1987). Bacterial overgrowth and mucosal changes in isolated (Thirty-Vella) ileal loops in rabbits. Effects of intraluminal antibiotics. *Laboratory Investigation*.

